# Sex-Specific Differences in Rodents Following a Single Primary Blast Exposure: Focus on the Monoamine and Galanin Systems

**DOI:** 10.3389/fneur.2020.540144

**Published:** 2020-10-15

**Authors:** Lizan Kawa, Ulf P. Arborelius, Tomas Hökfelt, Mårten Risling

**Affiliations:** Department of Neuroscience, Karolinska Institutet, Solna, Sweden

**Keywords:** anxiety, post concussive disorder, post traumatic stress disorder (PSTD), serotonin, neuropeptide, locus caeruleus, dorsal raphe (DR), noradrenaline

## Abstract

Most blast-induced traumatic brain injuries (bTBI) are mild in severity and culpable for the lingering and persistent neuropsychological complaints in affected individuals. There is evidence that the prevalence of symptoms post-exposure may be sex-specific. Our laboratory has focused on changes in the monoamine and the neuropeptide, galanin, systems in male rodents following primary bTBI. In this study, we aimed to replicate these findings in female rodents. Brainstem sections from the locus coeruleus (LC) and dorsal raphe nuclei (DRN) were processed for *in situ* hybridisation at 1 and 7 days post-bTBI. We investigated changes in the transcripts for tyrosine hydroxylase (TH), tryptophan hydroxylase two (TPH2) and galanin. Like in males, we found a transient increase in TH transcript levels bilaterally in the female LC. Changes in TPH2 mRNA were more pronounced and extensive in the DRN of females compared to males. Galanin mRNA was increased bilaterally in the LC and DRN, although this increase was not apparent until day 7 in the LC. Serum analysis revealed an increase in corticosterone, but only in exposed females. These changes occurred without any visible signs of white matter injury, cell death, or blood–brain barrier breakdown. Taken together, in the apparent absence of visible structural damage to the brain, the monoamine and galanin systems, two key players in emotional regulation, are activated deferentially in males and females following primary blast exposure. These similarities and differences should be considered when developing and evaluating diagnostic and therapeutic interventions for bTBI.

## Introduction

Blast-induced traumatic brain injury (bTBI) is particularly prevalent in active combat, although terror attacks are increasingly putting civilians at risk of similar brain injuries (1, 2). Of those with a positive TBI screen, the significant majority are mild in severity but may still confer increased risk of developing mood and anxiety disorders (3, 4). Research into such post-trauma sequelae has predominantly focused on male subjects, in animal and clinical studies alike. This is evidenced by figures from a review showing that of the 9,822 published studies available on TBI, only 40 described separate outcomes for each sex (0.004%) (5).

However, as more women have joined the armed forces and their roles have been considerably expanded (6), they are also at greater risk of suffering similar injuries. Additionally, civilian exposures are indiscriminate of sex. Parallel concerns have also been raised following sports-related concussions where, in fact, higher incidence rates of concussion and post-trauma complaints have been reported in females compared to males (7). This has raised concerns about women's health, especially since little is known about the potential effects of sex on acute and persistent symptomology complaints post-TBI, often termed post-concussive syndrome (PCS) (8, 9).

Of the limited literature comparing male and female outcomes post-TBI, there is little consensus. A comprehensive review of 18 studies found mixed results in male and female veterans (10). Seven studies reported that women are at increased risk of post-traumatic stress disorder (PTSD) compared to their male counterparts, while in four studies, women actually showed decreased risk, and another seven found no differences between the sexes (10). It should be noted that not all of these studies specified the type of trauma they investigated, nor did they adjust for pre-deployment factors, which may have influenced their findings.

In contrast, other studies looking at veteran health following deployment have found that depression is consistently more prevalent in female veterans (11–13). Furthermore, three of these studies also reported increased incidence of PTSD and substance abuse in the males (12). In the military population, PTSD symptoms have substantial overlap with PCS and are often concomitant with mild bTBI (14, 15). In the civilian population, the prevalence of PTSD and depression is twice as high in women as in men (16–18).

Information processing in the brain may also differ between the sexes, thus influencing the types of symptom onset (12). It has been claimed that males may be more likely to externalise stress and thus more prone to substance abuse or rage, while females may internalise stress, thus putting them at increased risk of anxiety/mood disorders (19).

Two monoamine systems have been implicated in stress: the noradrenergic locus coeruleus (LC) innervating virtually all regions of the central nervous system (20, 21) and the serotonergic dorsal raphe nucleus (DRN) giving rise to similarly extensive forebrain projections (22, 23). In fact, dysfunctions in monoamine neurotransmitters, noradrenaline (NA) (24–27) and serotonin, (5-hydroxytryptamine; 5-HT) (28–30) have also been associated with mood/anxiety disorders, such as depression. Several neuropeptides have also been implicated in the pathophysiology of mood and anxiety disorders; in this context and given its co-localisation with NA in the LC, and with 5-HT in the DRN (31), the neuropeptide galanin has been of particular interest (32–37). Galanin, through its three G-protein-coupled receptors, GalR1, GalR2 and GalR3, regulates homeostatic and motivated behaviours and modulates the activity of monoaminergic neurons including in the DRN and LC (38, 39).

The rapid conversion of an explosive into gas during a detonation, results in an immediate increase in atmospheric pressure, followed by a sudden drop. These extreme pressure changes can result in complex and distinct classifications of blast injuries: primary (a result of the pressure wave coming into contact with the head, causing a transient pressure increase and tissue deformation), secondary (caused by projectiles from explosive devices), tertiary (the high-velocity blast wind propelling persons or objects into the air causing subsequent collisions and injury or causing tissue shearing), quaternary (other injuries from the explosive effects such as burns), and even quinary blast injury (additional injuries or morbidities resulting from additives to explosives or other environmental contaminants) (40, 41). These injuries interact with many cellular and molecular processes including the aforementioned monoamine and peptide systems. The different components of a blast exposure can be dissected using separate experimental models, for example, using a blast system with body protection except for the head and support of the head to reduce the acceleration loading of tertiary blast (42, 43).

However, the presence and impact of this multifaceted disease can be difficult to delineate and understand in the clinical population, nor is it easy to replicate in a single model. Animal models are often used to recapitulate a specific part of the blast and afford researchers precise control of the environment and resulting injury. However, the use of animal models presents translational limitations to the clinical populations and even across animal models. It is therefore important to replicate findings across different models and sexes to ensure changes observed in specific systems are robust (44–46). Two of the most commonly employed models include the blast and shock tube. The former uses real explosives, while the latter uses compressed gas such as helium to produce a shock wave closely mimicking the profile of a blast.

We have previously reported on the effect of a single primary blast exposure on the behaviour of male rats and changes to the monoaminergic and galanin systems across various brain regions and time points using a blast tube (47, 48) and confirmed that these changes are robust in another model of primary blast TBI, the shock tube (49). These changes include transient, short-lasting elevation in NA levels in a number of forebrain regions and transcripts of its rate-limiting enzyme, tyrosine hydroxylase (TH), in the brainstem. These are coupled with changes in the 5-HT rate-limiting enzyme, tryptophan hydroxylase 2 (TPH2) also in the lower brainstem at 1 day post-exposure (48).

In this paper, we explore how some of these changes compare in female rats exposed to a single primary blast exposure. Thus, in the present study, we assess the expression of the rate-limiting monoamine biosynthetic enzymes TH and TPH2 in the LC and DRN, respectively, and galanin transcript levels in both regions, using *in situ* hybridisation (ISH). We used immunohistochemistry (IHC) to look for degenerating neurons, signs of axonal injury and blood vessel leakage in the forebrain. Moreover, we also analysed the serum of both male and female rats using enzyme-linked immunosorbent assay (ELISA) for some relevant markers.

## Materials and Methods

### Animal Groups and Manipulations

Sprague–Dawley rats, 10 males and 23 females (Taconic, Ry, Denmark), 10–12 weeks old, were used. All experiments were performed in accordance with the Swedish National Guidelines for Animal Experiments, and approved by the Stockholm Animal Care and Use Ethics Committee (Stockholm Norra Djurförsöksetiska Nämnd). Animals were housed in groups of three or four in Type IV MakrolonR plastic cages under standardised conditions (12 h light/dark cycle, lights on at 07:00; temperature of 22 ± 0.5°C; and 40–50% relative humidity). Food and water were provided ad libitum to the animals.

Two separate experiments made up this study: Experiment #1 consisted of ISH and IHC analysis. Here, the female rats were sacrificed at two post-exposure time points: 1 day = 6+5, and 7 days = 6+6 exposed and sham, respectively. Additionally, ISH findings from a previously published studies of male rodents were used for comparison (47, 48). Experiment #2 included several ELISAs with serum from female rats sacrificed at 1 day and 7 days post-exposure from Experiment #1, and serum from exposed and sham males sacrificed at 1 day post-exposure (*n* = 5, 5 exposed and sham).

### Exposure Conditions

Animals anaesthetised by 4% isoflurane inhalation (Janssen, Stockholm, Sweden) were placed in a rigid metallic holder, which protected the torso and prevented acceleration movements of the head relative to the rest of the body. The holder was subsequently mounted into a 1.5-m metal blast tube (43, 50, 51), with the rat placed in a transverse prone position at a distance of 1 m from an explosive charge (43). Five grams of Swedish army plastic explosive containing explosive m/46, 86% pentaerythritol tetranitrate and mineral oil was used per blast exposure with a Nonel ignition (Dyno Nobel Sweden, Nora, Sweden). The rats' left side was exposed to a single primary blast TBI caused by the overpressure from the detonation, with a peak pressure of 550 kPa and a duration of 0.2 ms.

The Clemedson blast tube is one of the few systems that employ real explosives instead of compressed gas and has been in use since the 1950s. It has been recently modified to better control for pressure wave-induced acceleration of the animals mounted into the tube. The parameters of the resulting primary blast wave have been thoroughly studied and reported in Davidsson et al. (40). At the pressure waves employed for this study, no bleeding has been detected from the airways with the body protection used. The primary pressure peak of this model is very short, and its shape is akin to the classical Friedländer curve, thus more representative of open-field detonations rather than those found in confined spaces with reflections (40).

### Blood and Tissue Collection

Animals were anaesthetised with isoflurane and then injected with 1.5–2.0 ml of pentobarbital. Blood was obtained via a cardiac puncture and centrifuged at 10,000 RPM for 15 min at 4°C. The supernatants were aliquoted, fresh-frozen and stored at −70°C until processing. Animals were then decapitated, and the brains were removed, placed on dry ice and stored at −70°C until processing. All blast exposures and tissue collections occurred in the morning between 8 a.m. and noon.

### *In situ* Hybridisation

All samples for ISH were processed as previously described in detail (47). Briefly, serial coronal, 14-μm-thick sections were cut using a Cryo-Star HM 560 M (MICROM International GmbH, Heidelberg, Germany) at the level of the LC (bregma −10.52 to −9.16 mm) and DRN (bregma −8.30 to −7.30 mm), coordinates according to Paxinos and Watson (52). Two sections were thaw mounted per slide and three slides per animal were processed for ISH. Method of selection of slides was random. Oligonucleotides complementary to rat TH (53), TPH2 (54) and galanin mRNA were labelled with deoxyadenosine 5′triphosphate α-P32 (Perkin Elmer, Boston, MA) at the 3′-end using terminal deoxynucleotidyltransferase (Table 1).

**Table 1:** Primers used for oligo *in situ* hybridization.

**Probe**	**Primers**	**Gene Bank accession no**.
TH	GCG CTG GAT ACG AGA GGC ATA GTT CCT GAG CTT GTC	NM_012740
TPH2	TCC TCC GTC CAA ATG TTG TCA GGT GGA TTC AGC GTC ACA ATG GTG GTC	NM_017139
Galanin	GGTGCACAGTGGGTGTGGTCTCAGGACTGCTCT ATGCCAGGCAGGCTGTCGAGGGCCCCGGCCTCT GTGCGGACGATATTGCTCTCAGGCAGGGGTACA CCCGAGCCCCAGAGTGGCTGACAGGGTTGCA ACCAACAGGAGCCAGGC	NM_017139

The optimal exposure time was determined by exposing the slides to imaging plates (BAS-SR Fujifilm, Tokyo, Japan). Slides were developed using D19 developer (Kodak) and AL-4 fixative (Kodak) and mounted in glycerol-phosphate. Dark-field photomicrographs were captured in a microscope (Nikon Eclipse E-600), connected to a digital camera (Digital Sight, U1; Nikon). The images were analysed according to the mean grey density (MGD) of the mRNA signal in the regions of interest (ROIs), using ImageJ 1.48 (NIH).

### Immunohistochemistry

Sections from the ventral and dorsal hippocampus were used to stain for degenerating neurons using Fluoro Jade B (FJ; Merck Millipore AG310, Darmstadt, Germany), β-amyloid precursor protein accumulation (APP, Life Technologies, 51-2700, dilution 1:400; Stockholm, Sweden) and leakages of blood vessels (using a secondary rat antibody, Jackson ImmunoResearch, 712-225-153, dilution 1:100; Suffolk, UK) as previously described in detail (48). Sections from experiments with a focal penetrating injury model were used as a positive control (43).

### Serum Analysis

The levels of a number of markers were assayed in the serum using ELISA. All assays were run in accordance to manufacturer's instructions. Progesterone (PROG) and estradiol (E2) levels were measured using Rat PROG ELISA Kit and Rat E2 ELISA Kit (both from CUSABIO, Nordic BioSite, Stockholm, Sweden) at 0.2 ng/ml and 40 pg/ml sensitivity, respectively. Each sample was diluted 1:2. Corticosterone (CORT) levels were measured using Abcam's Corticosterone ELISA Kit (BioNordika, Stockholm, Sweden) at a sensitivity of 0.3 ng/ml.

### Statistical Analysis

All statistical analyses were performed using GraphPad Prism version 6 (GraphPad Software, CA). For ISH studies, exposed and sham groups were evaluated with using ANOVA and followed by the Tukey *post-hoc* tests. In the LC, no left vs. right differences were observed, so these groups were collapsed in the analysis. Also, no difference between the female shams of the two different time points were found, so these groups were also collapsed. The MGD of each ROI was normalised to its corresponding sham level to clearly show increases and/or decreases in transcript level on sham levels.

For the CORT ELISA, male vs. female was evaluated using ANOVA followed by *post-hoc* tests. For weight, PROG and E2 levels, *t*-tests were performed to compare sham to exposed groups.

All data are presented as the mean ± SEM, with *F* and *t* values reported for statistically significant findings. The level of significance is depicted as follows: ^*^p < 0.05, ^**^p < 0.01, ^***^p < 0.001 and ^****^p < 0.0001.

## Results

### Body Weight and Changes in Serum Markers

**Figure 1:**
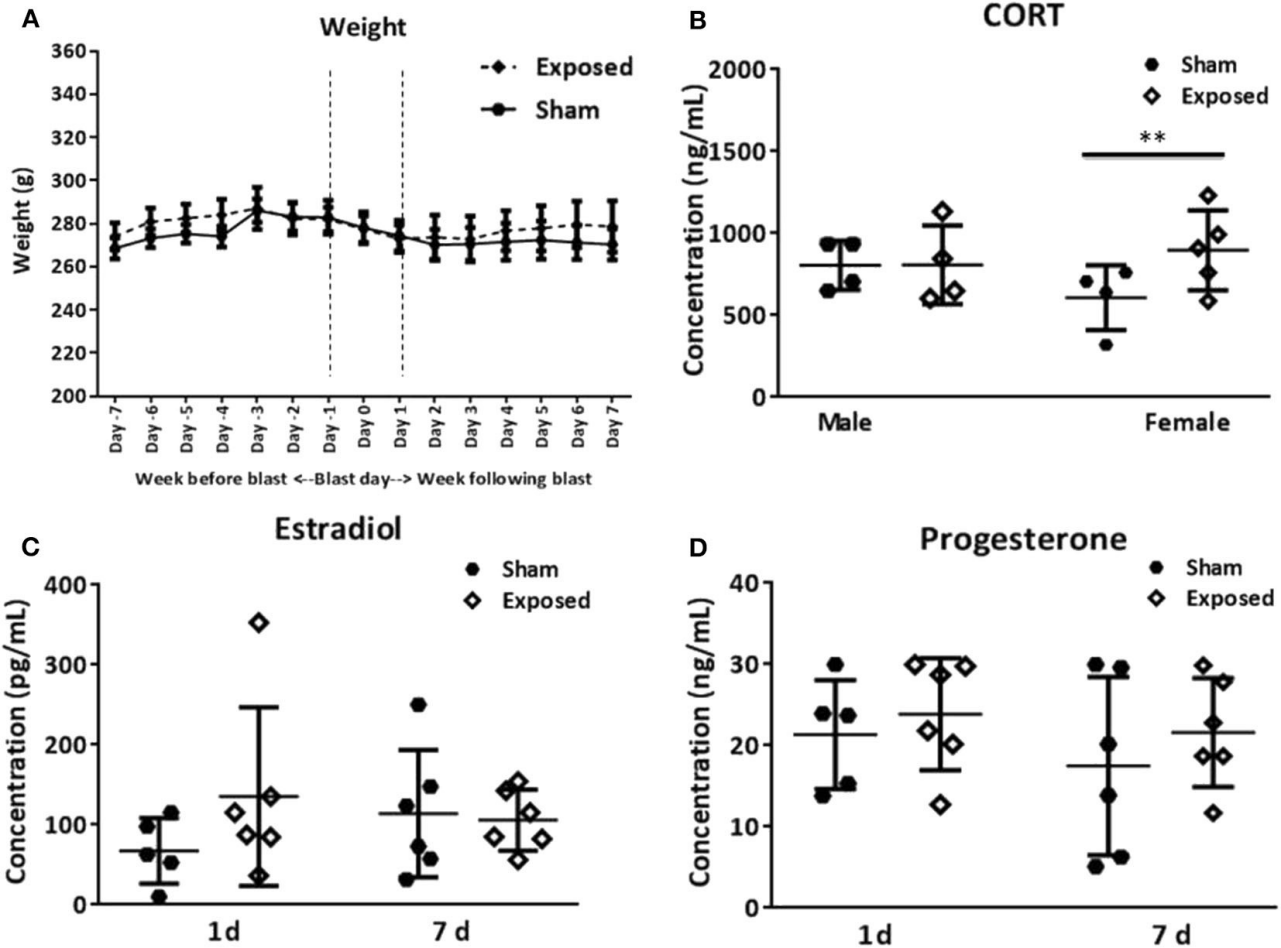
Basic parameters. Average weight of female rats, the week before and the week following blast exposure **(A)**. Serum analysis of CORT levels in female vs. males at 1 day following blast exposure **(B)**. The levels of the hormones estradiol **(C)** and progesterone **(D)** in females at 1 and 7 days as determined in serum using ELISA. No differences in body weight are encountered between the exposed and sham group. CORT levels are increased, but only in females. The female hormones vary among animals. CORT, corticosterone; ELISA, enzyme-linked immunosorbent assay. Data are presented as mean ± SEM. (^**^p < 0.01). Female *n* numbers included: at 1 day = 6 + 5, and at 7 days = 6 + 6 exposed and sham, respectively. For males sacrificed at 1 day post-exposure (*n* = 5, 5 exposed and sham). One of the shams from both the male and female group were excluded from the CORT analysis as they were outliers.

Female rats were weighed daily, a week before and after the primary blast exposure, and no significant differences in body weight were found between sham and exposed groups throughout the experiment (Figure 1A). The levels of the hormones estradiol and progesterone were measured from serum at the two terminal time points using ELISA. There were no statistically significant differences between sham or exposed groups, at either time point (Figures 1C,D). It should be noted that the concentration of either hormone did not allow us to determine the stage of the estrus cycle of the individual rats. The large spread in hormone levels across the groups likely indicates that the rats were at different stages in the estrus cycle. This large spread probably more closely mimics the clinical female population at risk of TBI.

CORT levels in the serum of female and male rats were assayed at 1 day post-exposure and elevated levels were found in the exposed relative to sham groups, but only in females (Figure 1B, *t* = 2.98, *p* < 0.01).

### Changes in Transcript Levels of TH, TPH2 and Galanin as Measured by ISH

**Figure 2:**
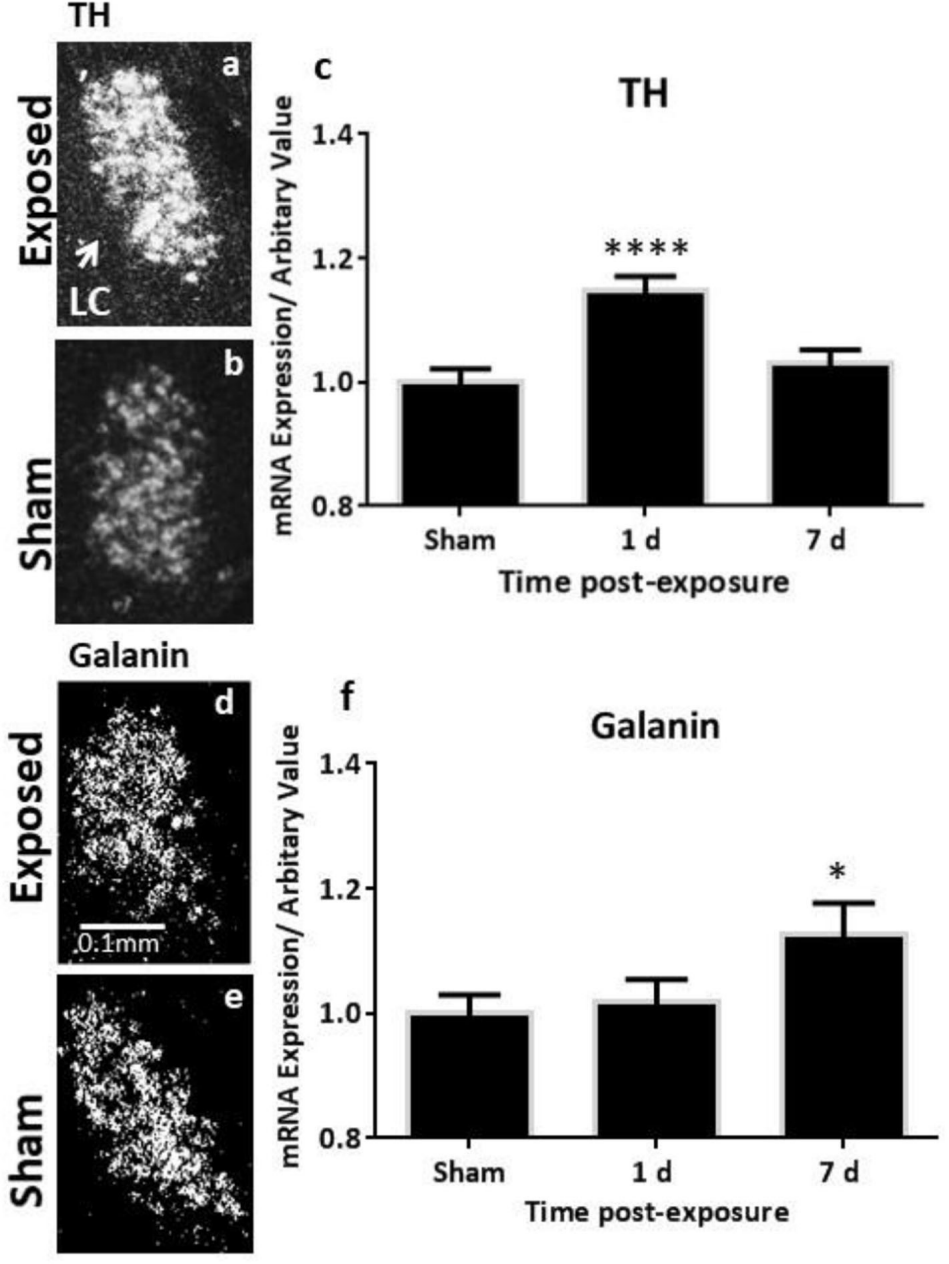
ISH analysis of transcript levels of TH and galanin in the LC following exposure to a single mbTBI in female rats. Representative dark-field ISH photomicrographs of emulsion-dipped sections showing the distribution and levels of TH mRNA in exposed **(a)** and sham **(b)** animals; galanin mRNA in the exposed **(d)** and sham **(e)** LC at 1 day post-exposure. Quantification of transcript levels indicates that TH mRNA levels are significantly increased bilaterally in the LC at 1 day and return to sham levels by 7 days post-exposure **(c)**. Galanin transcript only reaches statistically significant levels by day 7 post-exposure **(f)**. There were no differences in the left vs. right LC, so these groups were collapsed. The same is true for 1-day and 7-day shams, so these groups were also collapsed. All transcript levels have been normalised to their respective shams. ISH, *in situ* hybridisation; LC, locus coeruleus; TH, tyrosine hydroxylase. Data are presented as mean ± SEM. (^*^p < 0.05, ^****^p < 0.0001). Female *n* numbers included: at 1 day = 6 + 5, and at 7 days = 6 + 6 exposed and sham, respectively.

**Figure 3:**
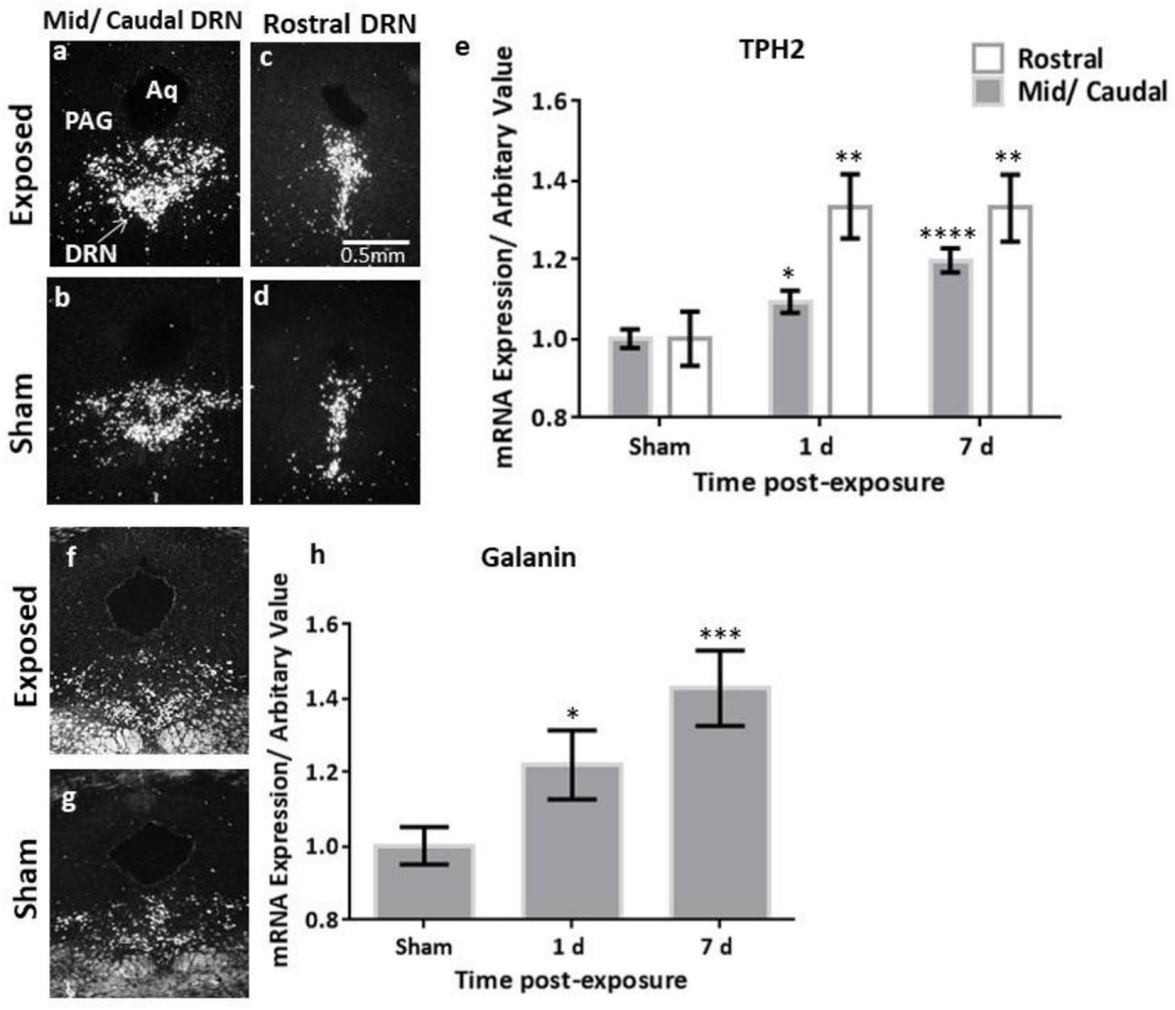
ISH analysis of transcript levels of TPH2 and galanin in the DRN following exposure to a single mbTBI in female rats. Representative dark-field ISH photomicrographs of emulsion-dipped sections showing the distribution and levels of TPH2 in the mid/caudal **(a,b)**, and rostral **(c,d)** DRN, and galanin in the mid/caudal **(f,g)** at 1 day post-exposure. Quantification of TPH2 mRNA in the DRN **(e)** indicate that TPH2 is significantly increased in both the mid/caudal and rostral DRN at 1 day post-exposure, and remains elevated even at 7 days post-exposure, relative to sham levels. Quantification of galanin mRNA in the DRN was only explored in the mid/caudal region **(h)** and is significantly elevated already at 1 day post-exposure, and remains elevated at 7 days post-exposure, relative to sham levels. The sham groups of the two different time points were not statistically significantly different, so these groups were also collapsed. All transcript levels have been normalised to their respective shams. Data are presented as mean ± SEM. (^*^p < 0.05, ^**^p < 0.01, ^***^p < 0.001, ^****^p < 0.0001). ISH, *in situ* hybridisation; DRN, dorsal raphe nucleus; TPH2, tryptophan hydroxylase 2. Female *n* numbers included: at 1 day = 6 + 5, and at 7 days = 6 + 6 exposed and sham, respectively.

**Figure 4:**
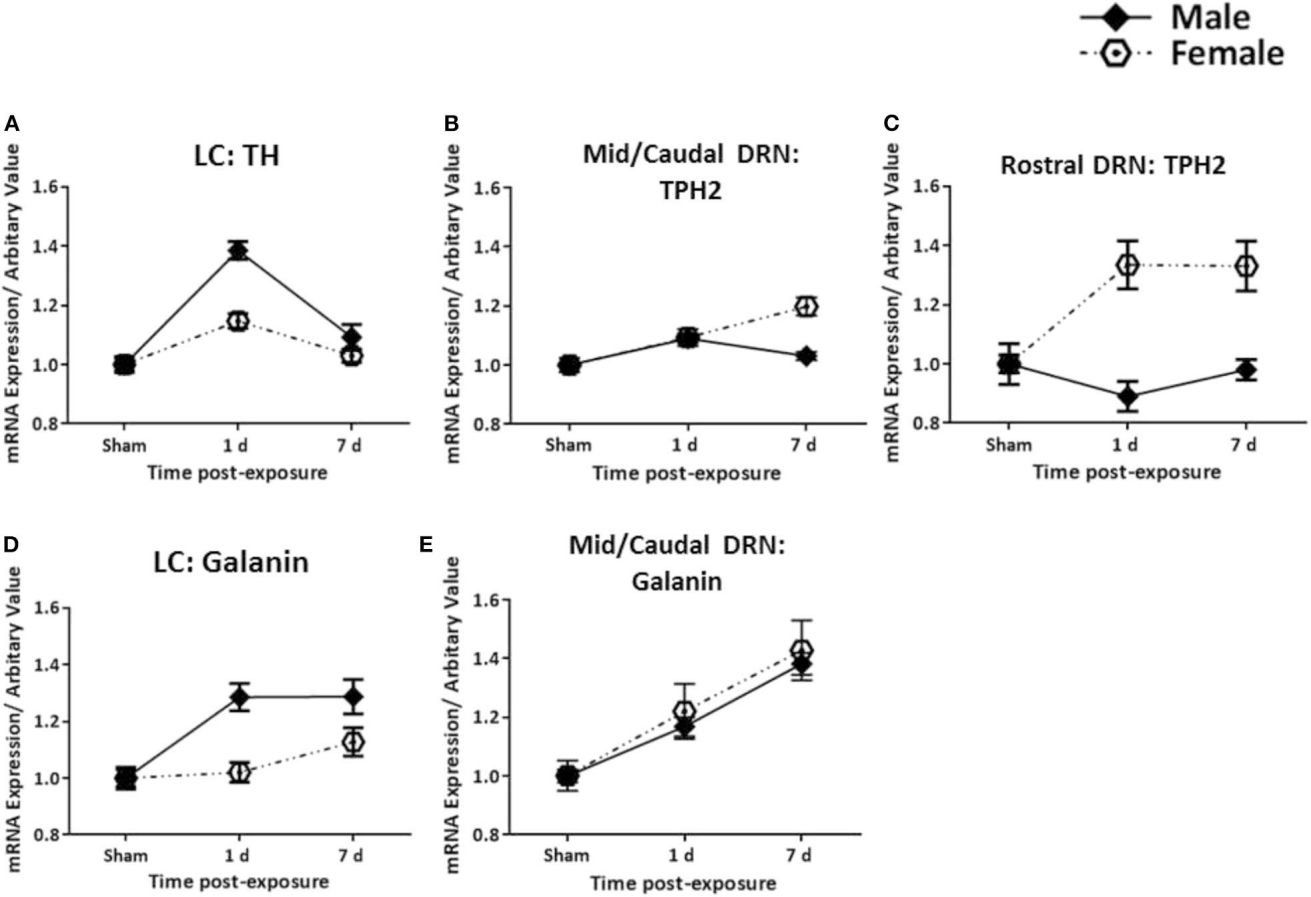
Comparison between female and male rats. Changes in transcripts for TH, TPH2, and galanin in females (present study) and for already published findings TH, TPH2, and galanin in males (47, 48) in the LC **(A,D)** and DRN **(B,C,E)** are shown. The increase in TH mRNA levels is more pronounced in males as compared to females, and is only seen at 1 day. The galanin transcript levels increase faster in males than females, but are still elevated at 7 days in both sexes. In the rostral DRN, TPH2 mRNA levels are increased at 1 and 7 days in females, sharply contrasting males. Galanin mRNA shows gradual and parallel increases in the Mid/Caudal DRN. All transcript levels have been normalised to their respective shams. LC, locus coeruleus; TH, tyrosine hydroxylase; DRN, dorsal raphe nucleus; TPH2, tryptophan hydroxylase 2.

The analysis of the TH transcript levels revealed a rapid and significant increase in female rats bilaterally in the LC (*F* = 11.73, *p* < 0.0001, Figures 2a–c). This normalised by 7 days post-exposure (Figure 2c), akin to our previous observations in the males (Figure 4A). The transcript levels of the key biosynthetic enzyme TPH2 were significantly increased at 1 day post-exposure, in both the mid/caudal (*F* = 13.62, *p* < 0.05) and rostral DRN (*F* = 6.52, *p* < 0.01, Figures 3a–e). These levels remained elevated even at day 7, in both the mid/caudal (*p* < 0.001) and rostral DRN (*p* < 0.01) of female rats (Figure 3e). While the acute findings in the mid/caudal part of the DRN in the females and males are alike (Figure 4B), in the females, increased transcript levels were already observed at 1 day and persisted at 7 days, the longest time point studied. They also extended to the rostral part of the DRN (Figure 4C).

While galanin transcript levels also increased in the LC (Figures 2d–f), this was slower and less pronounced than in males (Figure 4D), becoming only statistically significant at day 7 post-exposure (Figure 2f, *F* = 4.4, *p* < 0.05). In the DRN (Figures 3f–h), we only explored galanin transcript levels in the mid/caudal DRN, where it also slowly and steadily increased, but reaching statistically significant levels already at 1 day and continuing to increase 7 days post-exposure (*F* = 5.22, *p* < 0.05 and *p* < 0.001, respectively, Figure 3h). This finding resembles what is seen in male rats (Figure 4E).

### Degeneration, Blood Vessel Leakage and APP Accumulation Assessed by IHC

In sections from the dorsal and ventral hippocampal formation, exposed rats did not appear to have leakage in blood vessels in these forebrain regions, nor could cell degeneration be detected in any of these areas. Evaluation of the white matter tracts by staining for APP accumulation revealed no difference to the shams at either time point post-exposure (data not shown).

## Discussion

In this study, we show that exposure to a single primary blast wave results in both acute and longer-lasting changes in a sex-specific manner. We report a transient and acute increase in the catecholamine-related transcript TH in female rodents, similar to our previous observations in males (48). We also demonstrate an increase in serotonin-related TPH2, although this appears to be more pronounced and persistent in females. Changes in galanin transcript levels are slower and less pronounced in the female LC as compared to male rats but show the same trend for males and females in the mid/caudal DRN. Moreover, analysis of CORT reveals elevated levels in the serum of only exposed females relative to shams. These changes were seen, in the absence of detectable neuropathological changes, in concert with our previous observations in the males. These findings lend further support to the involvement of the noradrenergic, serotonergic and galanin systems in mild mbTBI and also emphasise the need to consider potential sex-specific differences.

### Mild TBIs and Sex Considerations

Recently, more women have been deployed to the current conflict zones than at any previous point in history, thus placing them at similar risk for combat-related injuries and health problems as their male counterparts (55). Traumatic brain injuries, as a result of exposure to explosive devices and associated post-concussive symptoms, have been widely reported in the literature, although the vast majority of studies have not considered sex-specific differences (8, 56, 57). Some studies have found that women are more likely than men to report post-concussive symptoms after a mild TBI, both in the civilian and military population (58–61). However, not all studies are in agreement; findings by Jackson et al. reveal no sex-specific differences in post-concussive symptom reporting and rates of PTSD or in probable major depression diagnosis in US veterans (62). Pugh et al. found similar comorbidity trajectories between the sexes, but differences within the trajectories. For example, females were more likely to have depression and headaches, whereas men were more likely to have back pain and substance use disorder (13).

### Sex Difference in Animal Models of TBI

Animal studies of TBI have shown that females are more resistant to TBI than males. For example, mRNA levels of cyclooxygenase-2 (COX-2), a pro-inflammatory enzyme, was found to be significantly more elevated in males compared to females, in a penetrating model of TBI (63). Here, COX-2 expression also correlated with increased apoptotic cell death, so females appeared to have better outcome following injury. In a follow-up study, blocking COX-2, using the established inhibitor diclofenac, Dehlaghi et al. saw decreased apoptosis in the perilesional area following focal penetrating TBI (64). While the reason for this sex difference in animal models is unclear, the female sex hormones are suggested to afford some neuroprotection to females (46). In particular, progesterone has been shown to have a plethora of neuroprotective effects following TBI in animal models, including inhibition of inflammation, reducing edema, enhancing re-myelination, and improving functional recovery (65–67). Evaluating studies based on injury severity revealed some interesting sex-specific differences. Specifically among studies of mild TBI, only 17% of studies showed better outcomes in females than males, while in moderate–severe TBI, a larger proportion (55%) showed better outcomes in females (68).

### Monoamine Transcripts

There is comprehensive evidence linking dysfunctions in the monoamine systems to mood and anxiety disorders such as depression. In animal studies, elevated levels of the transcripts TH and TPH2 have also been reported following exposure to various stressors (69–72). Tóth et al. explored changes in TH mRNA following chronic repeated restraint in A1 noradrenergic cell bodies in the brainstem. They found a 50% increase in TH mRNA levels in both male and female rats, 24 h after the last session (73). While our observations are generally in agreement with this study, we too see an increase in TH mRNA in both sexes, although the apparent increase is less dramatic in females than males, as shown in Figure 4. However, this difference should be noted with caution, since the present female findings are compared with results obtained in males in a previous study (48).

Changes in the LC have a direct impact on the noradrenergic terminals in the forebrain and on NA turnover (26). In fact, we have previously demonstrated increased NA levels in several forebrain regions including the prefrontal cortex and both the dorsal and ventral parts of the hippocampal formation in the same model (48). This translated to an increase in climbing behaviour in the forced swim test, which we interpreted as hyperarousal, based on previous literature (74).

Variations in TPH2 genetic transcripts and expressions have also been linked to a number of disorders including PTSD, depression, and panic attacks (75–77). Dysfunctions in TPH2 are likely to influence serotonergic functioning and thus play a role in the pathogenesis of emotional and cognitive disorders, given the wide functions of 5-HT (29, 78–81). We found a more pronounced and persistent increase for TPH2 in both the mid/caudal and rostral DRN in females. Changes in males were more modest, limited to the mid/caudal DRN and only elevated acutely post-exposure (Figure 4). Given some of the clinical reports in veterans, e.g., the increased prevalence of depression or PTSD with comorbid depression in females, our findings here seem relevant.

The DRN is a more complex region with regard to chemical neuroanatomy than the LC. Also, this region is sensitive to estrogen, via estrogen receptor ß, and the interplay with stress on TPH2 expression (82). The DRN is composed of multiple, functionally distinct sub-regions that receive anatomically distinct inputs (83), and these sub-regions vary in their expression of estrogen receptors in rats (84). Hiroi et al. explored the interaction of estrogen and TPH2 expression in the caudal DRN on anxiety-like behaviour in ovariectomised rats (76). They found that rats given estradiol capsules in conjunction with virally induced overexpression of TPH2 were anxiogenic. Thus, animals spent significantly increased time in the corners of the test, while either treatment alone significantly increased time spent in the center of the open field, indicative of decreased anxiety (76). Given our female rats were at various stages of the estrus cycle as evident by the large variance in estradiol and progesterone levels in the ELISAs, we cannot determine what contributions either of these hormones may have had on these different findings across the two sexes.

### Galanin Transcripts

The changes in galanin in the DRN were, in principle, similar to those recorded in males. However, in the female LC, the increase in galanin transcript was slower and less pronounced than in males, reaching statistical significance only at 7 days post-exposure. Whether or not this reflects the abovementioned higher resistance of females than males to TBI (63) remains to be determined, as does a possible neuroprotective role of female sex hormones (46).

Galanin was originally cloned from an estrogen-induced pituitary tumour cDNA library (85, 86). In some brain regions, galanin expression is sensitive to estrogen (87). Tseng et al. have studied ovariectomised rats chronically treated with estrogen and shown that galanin, but not TH gene expression, is regulated by estrogen (88). Interestingly, in a study using postmortem brains from depressed subjects who committed suicide and relevant controls, the galanin levels were significantly higher only in the LC of the depressed women, and not in the males, nor in four other brain regions (89). These results suggest that galanin expression in the LC in females is selectively sensitive to sex hormones and perhaps varies across species. Galanin expression in the LC has been associated with resilience to depression (33, 90). To what extent the changes in galanin observed here could have a similar function remains to to be analysed. It should be noted that our results are obtained during the first week after stress/blast, whereas the Barde et al. (89) study examines brains of subjects who had been ill for a long time.

A number of studies have shown that stress can change the expression of galanin (91, 92). Electrophysiological, behavioural and neurochemical studies have shown that galanin exerts modulatory (mainly inhibitory) effects on both the noradrenergic and serotonergic systems (35, 37, 93). The potential auto-inhibitory role on LC neurons may be an important mechanism in offsetting the increased NA release following stress (33).

The action of galanin is mediated via three G protein-coupled receptors, GalR1–GalR3 (38, 94). Among these receptors, GalR1 and GalR3 mainly activate Gi/o types of G proteins mediating inhibitory actions of galanin, while the GalR2 subtype can, among others, transmit stimulatory effects of galanin. Variability in the modulation of these circuits and transmitters involved may be a reason for contradictory results.

### Depression and Inflammation

Associations between stress exposure and activation of the inflammatory response have been reported. Cernak (95) showed that there is a systemic inflammatory response to blast exposure, which includes the brain, and elevated CORT, interferon-γ (IFN-γ), and interleukin 6 levels have also been found in animals exposed to a blast overpressure (96). Emerging data implicate the inflammatory response following stress exposure in the pathobiology of depression [see Miller and Raison (97)]. It is postulated that following stress, NA release can start a signaling cascade that includes activation of pro-inflammatory cytokines that may then impact the availability of NA, 5-HT, and dopamine in the brain. Many of the cytokines can also reduce the availability of monoamine precursors such as tryptophan and reduce the synaptic availability of these monoamines, a hallmark of depression.

Overall, CORT levels in both the female and male sham groups are also quite high. This may be attributed to the experimental conditions, where all animals are kept in the same room, and while only the exposed animals are injured, the shams are still exposed to all other experimental manipulations. This includes the blast acoustics, handling, and anaesthesia. Others have reported on factors beyond the blast parameters inducing physiological changes in animals. For example, Kamnaksh et al. (96) detected elevated CORT, interferon-γ, and interleukin 6 levels in sham animals relative to naïve animals, not exposed to any stressors (96, 98). The elevated CORT findings are interesting in light of these emerging themes, particularly as it was only statistically significant in the exposed females.

Studies in animals and humans consistently report sex-specific differences in baseline anxiety levels, response to intense stressors, and even how these stressors may be acquired (99, 100). Females are reported to have enhanced glucocorticoid reactivity, as well as resting and stress-induced hypothalamic–pituitary–adrenal axis activation (4). In a study by Xing et al., the females had more elevated CORT levels than males even 3 weeks after chronic mild stress exposure (101). In another study, females with no previous history of mental illness, in general, showed higher anxiety scores than males in the Hospital Anxiety and Depression Scale (HADS) (99). We have evaluated changes in our exposed animals against shams. Given the already heightened basal levels and stress response in females, perhaps the additive impact of the primary blast exposure is difficult to evaluate in already activated systems, especially ones that are as sensitive as the LC. This might explain the modest increases in the exposed vs. sham levels of TH and galanin transcript levels in the LC of the females. However, it also highlights the changes in systems that were robustly upregulated such as TPH2 in the DRN.

### Limitations

There are limitations of our work, particularly regarding the ELISAs, where there can be concerns regarding poor reproducibility between laboratories and sensitivity issues. Being unable to ascertain the stage of the estrus cycle the rats in the ELISA studies has drawbacks. Other methods such as vaginal smears would have given us a more accurate picture. Furthermore, exploring CORT levels at additional time points post-exposure could be more informative than just a snapshot.

The longest time studied in this experiment was 7 days, where galanin and TPH2 mRNA levels were at their peak levels in exposed females. It would be interesting to define an end point when transcripts return to sham levels, i.e., how long-lasting are these elevations after the bTBI? Finally, running both male and female studies in the same experiment would have increased the possibility to make a direct comparison between groups.

## Concluding Remarks

All pharmacotherapies thus far developed for TBI have failed. Some of these failures may be attributed to translational challenges arising between experimental models and the clinical population. These shortcomings include the differing time scales of rodent and human pathological processes, the impact of physical and biomechanical forces on the rodent vs. human brain, and the lack of reproducibility of findings across models, species or sex (44–46). However, as the relevance of this area extends beyond the military and war zones, to civilian accidents, such as the recent catastrophic blast in the city of Beirut, progress in this area is even more pressing.

We have previously explored these changes in males, both in the monoamine and galanin systems, at multiple time points post-exposure, including single and repeated exposures (47, 48). The changes obtained in these studies have been further confirmed by results obtained in a different primary blast model, the shock tube, which uses compressed gas in place of explosives and has a slightly different peak pressure and duration (49). Here, we present findings that the same systems are perturbed in females, even if interesting differences were encountered. There is therefore strong and confirmatory evidence to support that in the absence of cell death or other signs of classical neuropathology, the changes in the NA, 5-HT, and galanin systems are robust across models and sexes. These systems are likely involved in a cascade of neurochemical changes following mild bTBI and could be an important component in the pathophysiology of primary blast injuries. Progress in potential interventions and therapeutics should consider these systems and possible sex-specific differences.

## Data Availability Statement

The datasets generated for this study will not be made public because the information is not in a readily available format to be shared. Further inquiries can be directed to the corresponding author.

## Ethics Statement

The animal study was reviewed and approved by Stockholm Animal Care and Use Ethics Committee (Stockholm Norra Djurförsöksetiska Nämnd).

## Author Contributions

MR, TH, and LK contributed to the design of the study. LK performed all experiments and the statistical analysis and wrote the first draft of the manuscript. UA performed the blast exposures. All authors contributed to manuscript revision, read, and approved the submitted version.

## Conflict of Interest

TH has shares in Bioarctic and Lundbeck. The remaining authors declare that the research was conducted in the absence of any commercial or financial relationships that could be construed as a potential conflict of interest.
